# Investigating the Effectiveness of an Educational Escape Game for Increasing Nutrition-Related Knowledge in Young Adolescents: A Pilot Study

**DOI:** 10.3389/fnut.2021.674404

**Published:** 2021-05-28

**Authors:** Anna M. Abdollahi, Natalie A. Masento, Henna Vepsäläinen, Michal Mijal, Monika Gromadzka, Mikael Fogelholm

**Affiliations:** ^1^Department of Food and Nutrition, University of Helsinki, Helsinki, Finland; ^2^Department of Food and Nutritional Sciences, University of Reading, Reading, United Kingdom; ^3^Faculty of Management, University of Warsaw, Warsaw, Poland

**Keywords:** escape game, educational game, game-based learning, nutrition education, nutrition knowledge, school-aged children

## Abstract

**Objective:** As a pilot trial under the Games of Food consortium, this study assessed the effectiveness of an educational escape game alongside a self-study method as a nutrition knowledge intervention. Furthermore, this study explored the use of an escape game as an educational tool for young adolescents.

**Materials and Methods:** Altogether three schools participated, one from Finland and two from the UK. Baseline questionnaires assessing knowledge were administered before intervention day. Participants from each class were randomly allocated by the researchers into either the escape game condition, where participants played a nutrition education escape game with a focus on plant-based protein sources, or the self-study condition, where participants received an educational leaflet with identical content. In addition to the knowledge post-assessment, the educational escape game condition answered an enjoyment and intrinsic motivation questionnaire to evaluate the game experience. Paired *t*-tests were used to determine significant changes within intervention conditions and ANCOVA was used to estimate the differences in knowledge.

**Results:** The participants were 130 children (11–14 years), divided into educational escape game (*n* = 68) and self-study (*n* = 62) conditions. Both the educational escape game (20.7 vs. 23, *p* < 0.001) and self-study (21.1 vs. 23.1, *p* = 0.002) had improved overall knowledge scores. No significant differences in gained knowledge existed between groups. Of the educational escape game participants, 60% reported the game as mostly enjoyable and 46% reported added use and value for learning.

**Conclusion:** The educational escape game condition was comparable to the self-study method for nutrition education in adolescents. However, since the educational escape game provides an enjoyable experience that may enhance intrinsic motivation to promote learning and possible behavior change, the use of escape games for nutrition education warrant further investigation.

## Introduction

The contribution of diet to health and wellbeing is irrefutable. A healthful diet is particularly important for young adolescents, who are in a crucial pubertal phase of development and growth (1). Though many adolescents do not follow recommended dietary guidelines (2–4), many exceed protein intake recommendations and have high consumption of animal-based protein, which has been associated with higher weight status (5). Lasting diet patterns and food preferences are formed during childhood, therefore targeting youth at an age where autonomy in food choice is reachable would further promote healthy dietary habits into adulthood (6, 7). Effective intervention strategies to reverse the growing trends in childhood obesity and improve diet quality remain high priorities in public health (8).

Increasing nutrition knowledge and interest are typical strategies for nutrition intervention and important prerequisites for eliciting diet-related behavior changes (9–13), though their impact may be weak (11). Activities that improve food literacy, which encompasses nutrition knowledge, have shown to be effective in improving dietary behaviors among adolescents (12, 13). Nutrition knowledge can include a plethora of different concepts, not all of which are relevant for adolescence in the general population. Previous studies have varied in their inclusion of nutrition knowledge concepts and methods of measuring change in knowledge (8, 12–14), highlighting the need for further investigation to explore how different interventions can support different concepts of nutrition knowledge in varied age groups. Educational games have shown success in health promotion, with meta-analyses finding positive effects in knowledge, attitude, behavior, and biological indicators from board games (15, 16), as well as digital games (16, 17). Though many educational game results go unpublished (18), studies evaluating specific games for nutrition education have shown improvement in game-related knowledge immediately (19, 20) and up to 1 year after gameplay (21) among young adolescents. Game-based education may enhance learning opportunities by positively affecting motivation and interest of participants (18, 22, 23). A recent review found that game-based education, especially those with compelling and immersive storylines, can have an impact on children's eating behaviors (16).

Escape games are once-played, team-based games that physically immerse players in a narrative to reach an ultimate goal (typically escape), by solving standard game mechanics within time-pressured environments. The challenges of an escape game make for memorable and rewarding game-play, which are elements particularly suited for behavior change directives (16, 24).

Escape games have become popular over the last decades, with educational escape games recently gaining more traction. Nevertheless, only a handful of studies on the effectiveness and efficacy of educational escape games exist, and all have been conducted in adult populations. Studies have reported both increased (25–27) and decreased (28) knowledge scores after gameplay. Furthermore, all studies lack comparison with traditional or passive learning methods, such as self-study. Remarkably, the handful of existing adult studies utilizing educational escape games all report positive feedback in learning and enjoyment (25–29).

The lack of consistent evidence of the effectiveness of educational escape games as mean to increase in knowledge warrants further research. Though commercialized applications of (digital and physical) educational escape games in primary and secondary education classrooms exist, to this day there are no peer-reviewed publications on either nutrition interventions or school-aged children regarding the effectiveness of educational escape games.

The aim of this intervention trial was to pilot the assessment and compare the effectiveness of the educational escape game, Zombie Attack, with self-study material for increasing short-term nutrition knowledge of 11–14 year old children. Another aim was to identify if an educational escape game was an appropriate pedagogical tool for school-aged children.

## Materials and Methods

This binational pilot study was conducted in 11–14 year old children from Helsinki, Finland and Reading, United Kingdom (UK) as a part of a larger project, Games of Food (www.gameoffood.com). Intervention materials were designed by the partners of the Games of Food consortium, which comprises of researchers and developers from Technion Institute in Israel, University of Warsaw in Poland, University of Reading in UK, University of Helsinki in Finland, and the European Food Innovation Commission (EUFIC) in Belgium. The project was financially sponsored by EIT Food, a subset of the European Institute of Innovation and Technology (EIT) funded by the Horizon 2020.

Two school classes from Finland were recruited based on teacher interest and an invitation to participate was provided for every subsequent student and guardian. The participants from Finland consisted of an eighth (12–13 years old) and ninth (13–14 years old) grade class from a school specializing in physical education. Two schools from UK, with classes from seventh (11–12 years old) and eighth (12–13 years old) grades participated. All participants were randomly allocated within their classes to participate either in the educational escape game or the self-study condition. In Finland, students conducted the intervention conditions in separate rooms, with only one team in the educational escape game condition room at a time. In the UK, intervention conditions for each class were conducted simultaneously within a large gymnasium, where students were spatially separated. Background data, as well as baseline knowledge, were assessed prior to intervention periods. The background questionnaire inquired whether or not participants had previous experience with escape rooms or any dietary restrictions, along with basic demographic information, including age, gender, and date of birth.

All students that had provided written informed consent and written parental informed consent were eligible to participate. The study was reviewed by the Ethical Review Board in the Humanities and Social and Behavioral Sciences of the University of Helsinki (statement 10/2019) and the University Research Ethics Committee of the University of Reading (statement 19/20).

### Interventions

Both educational escape game and self-study material presented key information on healthy diet with a focus on plant-based protein intake. Educational content centered around nine nutrition topics: (1) food pyramid, (2) macronutrients, (3) food energy calculation, (4) protein recommendations, (5) essential amino acids, (6) complete proteins, (7) complementary proteins, (8) functions of protein in the body, and (9) sustainable sources of protein-rich foods. Nutrition researchers from the University of Helsinki reviewed the content for nutrition education.

#### Educational Escape Game Condition

The escape game Zombie Attack is a portable game designed to engage players with a post-apocalyptic storyline that is meant to create a sense of alarm and urgency during gameplay. The players are encouraged to work together as a team to save themselves and humanity by learning about a healthy and sustainable diet, as it is the only repellent against the zombie attacks. Each of the nine linear puzzles focuses on one aforementioned nutrition topic and plays a role in the elaborate storyline. Puzzle mechanics incorporate matching, padlocks, cipher, videos, and puzzles that require players to calculate energy, identify correct food sources, read nutrition labels, and evaluate recipes. Zombie Attack was developed in English and later translated into the Finnish language. Posters and infographics unrelated to the puzzle mechanics were placed around the team to maximize exposure to the nutrition education content throughout the gameplay.

Teams of 3–5 players were drawn within the participating classes. The interventions were allocated for 1 h. Study researchers supervised the gameplay and provided technical advice to teams only when necessary for game progression.

#### Self-Study Condition

The participants in the self-study condition were each provided material in leaflet form with the same nutritional messages as in the educational escape game. In Finland, each participant individually read the material of the intervention. In the UK, participants were individually given copies of the leaflet to read alone, but were allowed to sit and discuss in groups of 3–5 children.

### Outcome Measures

#### Nutrition Knowledge

A questionnaire assessing knowledge concerning nutrition concepts was administered ~1 week before the intervention sessions (baseline questionnaire), and immediately repeated after the intervention (post questionnaires). Questionnaires were created based on previously validated questionnaires from the General Nutrition Knowledge Questionnaire-revised (30) and a nutrition knowledge questionnaire for young athletes (31). Questions selected were, if necessary, altered to reflect the learning objectives relevant to healthy diet that were appropriate for the age group participating in the study with emphasis on plant-based proteins. The knowledge questionnaire consisted of 41 nutrition-related multiple choice and true or false questions. Knowledge scores were evaluated by summing 1 point for every correct answer and none for “unsure” or incorrect answers. The knowledge questionnaire was divided by topic for sub-analysis based on the centralized topics of the nutrition knowledge questionnaire: protein (14 questions), sugar (8 questions), fiber (9 questions), and reduced disease risk (10 questions).

#### Intrinsic Motivation

All educational escape game participants received an additional post intervention questionnaire to evaluate the game. The 14-item questionnaire, adapted from the intrinsic motivation inventory (IMI) (32), consisted of two 7-point Likert-scale (1 = not at all true, 7 = very true) domains based on the original IMI interest/enjoyment and value/usefulness subscales. This two-part questionnaire was used to assess enjoyment and intrinsic motivation, the latter which was defined as the usefulness and added value to one's self as perceived from the escape game.

### Statistical Analyses

Participants who completed baseline questionnaires and post knowledge questionnaires were used in the analysis, with the exception of the intrinsic motivation questionnaire analysis that included four additional participants who completed the educational escape game intervention without baseline data. Chi-square tests (categorical variables) and *t*-tests (continuous variables) were used to test significance between intervention conditions in baseline data. Paired *t*-tests were used to determine significant changes within intervention conditions. Differences in knowledge between intervention conditions were compared using analysis of covariance, which adjusted for baseline knowledge, age, and gender. Further adjustment for previous participation in an escape game and adhering to a special diet did not appreciably change the results. Internal consistency of knowledge subdomains, enjoyment, and intrinsic motivation were analyzed by Cronbach's alpha. Two-tailed *P* < 0.05 were considered statistically significant. All data was analyzed using free statistical software R (R Foundation for Statistical Computing, Vienna, Austria. ISBN 3-900051-07-0, http://www.R-project.org/).

## Results

**Figure 1:**
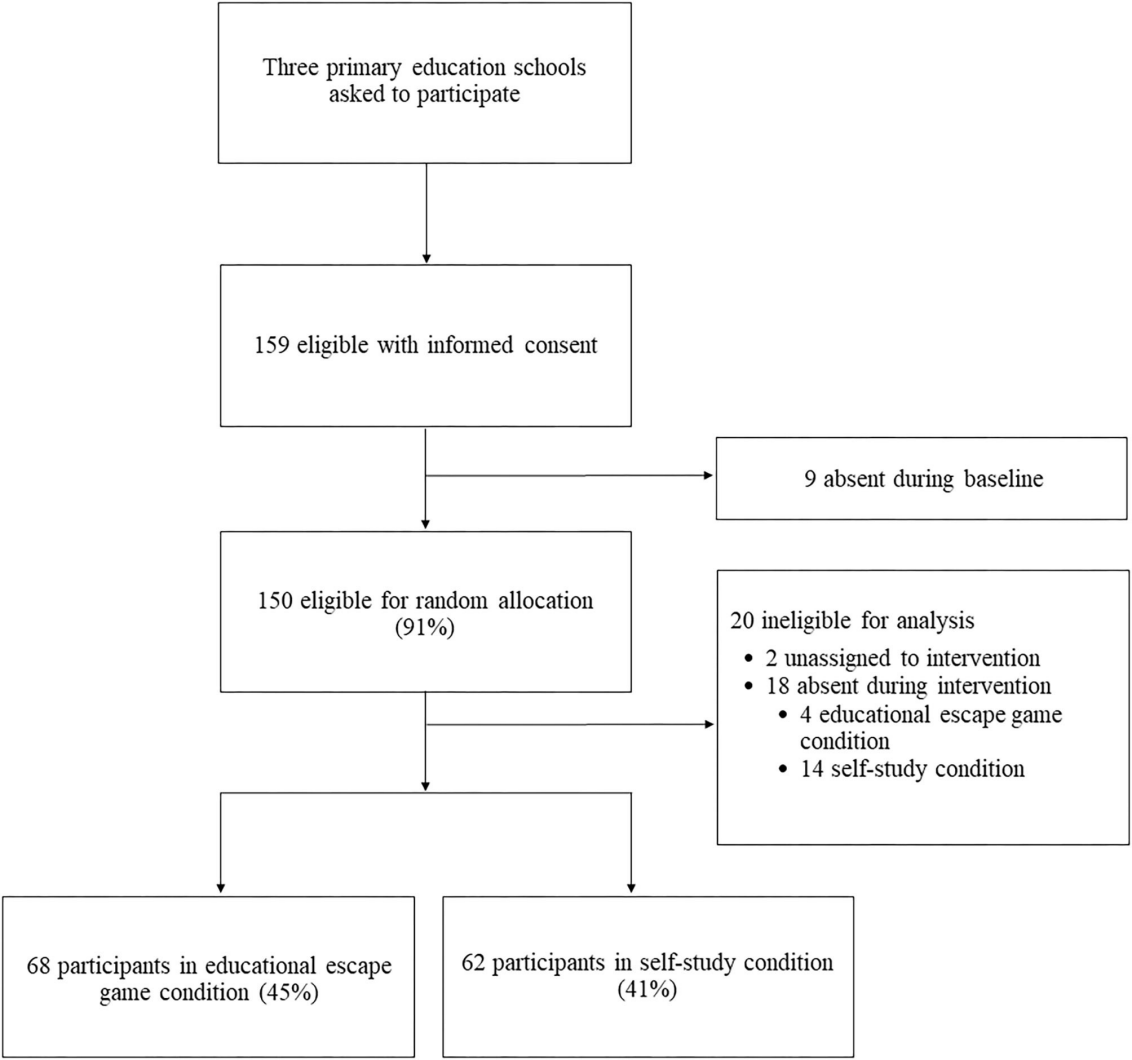
Flow diagram of study participants.

A total of 159 school-aged children were enrolled in the trial, of which 82% completed baseline and post-nutrition knowledge questionnaires (Figure 1). Of those present for both baseline and intervention days (*n* = 130), the mean age was 13.3 years (SD 1.2) and 81% were girls without significant differences between baseline nutrition knowledge, previous escape game experience, or adherence to a special diet between the educational escape game and self-study intervention conditions (Table 1).

**Table 1:** Baseline data by intervention condition.

	**Educational escape game (*n* = 68)**	**Self-study material (*n* = 62)**	**Total (*n* = 130)**
	total *N*(%) or mean ± SD	total *N*(%) or mean ± SD	total *N*(%) or mean ± SD
Age (years)	13.24 ±1.24	13.16 ± 1.20	13.25 ± 1.22
**Gender**			
Girls	55 (82.1%)	57 (81.4%)	105 (80.8%)
Boys	12 (17.9%)	13 (18.6%)	25 (19.2%)
**Country**			
United Kingdom	38 (64.2%)	48 (68.6%)	84 (64.6%)
Finland	24 (35.8%)	22 (31.4%)	46 (35.4%)
Participants with previous escape game experience	18 (27.3%)	8 (12.9%)	25 (19.2%)
Participants with a special diet	12 (18.2%)	19 (30.2%)	29 (22.3%)
Baseline nutrition knowledge^1^	20.69 ± 6.55	21.07 ± 7.07	20.87 ± 6.78

1 *Number of correct answers from 41-question nutrition knowledge questionnaire*.

Over half of the study participants were from UK (65%), with mean age (12.4 ± 0.4) and knowledge score (18.9 ± 6.8) in UK considerably lower than the age (14.8 ± 0.6) and knowledge score (24.5 ± 4.9) among participants from Finland. From UK, 98% of participants were girls, and in Finland 50%. From participants that completed both baseline and post questionnaires, 2.5% of knowledge questions in baseline and 0.8% in post questionnaires were left unanswered.

Improvement between baseline and post intervention nutrition knowledge average score significantly improved within both educational escape game [2.27 points, 95% CI (0.96, 3.57), *p* < 0.001] and self-study [2.03 points, 95% CI: (0.81, 3.25), *p* = 0.002] conditions. However, there was no significant difference in overall nutrition knowledge gained between the two intervention conditions (Table 2). Analysis of knowledge subdomains observed the participants in the educational escape game condition to have less improvement in post-intervention questions related to protein compared to the self-study condition participants, with differences between conditions estimated as −0.82 points [95% CI: (−1.56, −0.08), *p* = 0.03]. Conversely, educational escape game participants had a better improvement in fiber-related questions compared to the participants in the self-study condition, with differences between conditions estimated as 0.63 [95% CI: (0.09, 1.17), *p* = 0.02]. No differences between conditions were observed in sugar and reduced disease risk knowledge subdomains. Internal reliability of knowledge subdomains was moderate in sugar (α = 0.63) and fiber (α = 0.69), and moderately high in protein (α = 0.77) and reduced risk of disease (α = 0.80).

**Table 2:** Comparison of mean baseline and post-intervention knowledge scores in educational escape games and self-study intervention conditions.

		**Educational escape game (*****n*** **= 68)**		**Self-study material (*****n*** **= 62**		**Estimated difference^1^ (95% CI)**	***P*-value**
**Knowledge outcome**	**Number of Questions**	**Baseline**	**Post-intervention**	**Baseline**	**Post-intervention**		
Overall nutrition knowledge	41	20.69 ± 6.55	22.96 ± 7.09	21.07 ± 7.07	23.09 ± 7.63	0.08 (−1.63, 1.78)	0.93
Sugar	8	4.77 ± 1.53	5.78 ± 1.92	4.86 ± 1.34	5.79 ± 1.79	0.05 (−0.49, 0.60)	0.85
Fiber	9	4.10 ± 1.79	4.84 ± 1.87	4.44 ± 1.96	4.40 ± 1.99	0.63 (0.09, 1.17)	**0.02**
Protein	14	6.54 ± 2.69	7.06 ± 2.78	6.15 ± 2.73	7.57 ± 3.06	−0.82 (−1.56, −0.08)	**0.03**
Reduce disease risk	10	4.84 ± 2.26	6.12 ± 2.61	5.24 ± 2.58	6.23 ± 3.03	0.18 (−0.54, 0.89)	0.63

1 *Estimated difference between adjusted means of educational escape game and self-study post intervention scores. ANCOVA model adjusted for baseline knowledge, gender, and age. Statistically significant (p < 0.05) values are in bold*.

All educational escape game participants (*n* = 72) were able to escape within the allocated hour and generally reported high (mean ± SD = 5.8 ± 1.1) enjoyment with questions from a Likert 7-point scale, with mean interquartile range between 5.1 and 6.8. A majority (60%) of participants reported the game as mostly or completely enjoyable and 32% somewhat enjoyable. Questions assessing intrinsic motivation from usefulness and value found respondents to perceive the game as beneficial (5.5 ± 1.2), with mean interquartile range between 4.4 and 6.5. Slightly less than half (46%) of the participants reported the game as mostly or completely useful and valuable, followed by 44% reporting the game as somewhat useful and valuable (see Table 3).

**Table 3:** Assessment of enjoyment and intrinsic motivation questionnaire items of all educational escape game condition participants (*n* = 72).

**Domains and questions^1^**	**Not true**	**Somewhat true**	**True**	**Mean score SD**	**Cronbach's Alpha**
Enjoyment total	8%	32%	60%	5.81 ± 1.09	0.88
I enjoyed doing this activity very much	7%	31%	62%	6.14 ± 1.03	
This activity was fun to do	3%	42%	55%	6.01 ± 1.24	
I thought this was a boring activity (answer reversed)	3%	20%	77%	6.35 ± 1.17	
This activity did not hold my attention at all (answer reversed)	10%	21%	69%	5.98 ± 1.56	
I would describe this activity as very interesting	7%	43%	50%	5.77 ± 1.27	
I thought this activity was quite enjoyable	13%	30%	57%	5.86 ± 1.45	
While I was doing this activity, I was thinking about how much I enjoyed it	15%	37%	48%	5.23 ± 1.70	
Intrinsic motivation total	10%	44%	46%	5.45 ± 1.24	0.91
I believe this activity could be of some value to me	10%	49%	41%	5.38 ± 1.37	
I think that doing this activity is useful for learning about nutrition and health	14%	37%	49%	5.44 ± 1.66	
I think this is important to do because it can teach me about nutrition but also allows me to have fun	7%	41%	52%	5.75 ± 1.39	
I would be willing to do this again because it has some value to me	8%	36%	56%	5.74 ± 1.44	
I think doing this activity could help me to think about what foods I choose to eat	15%	47%	38%	5.20 ± 1.63	
I believe doing this activity could be beneficial to me	8%	51%	41%	5.49 ± 1.42	
I think this is an important activity	8%	45%	47%	5.54 ± 1.41	

1 *Based on Likert scale 1–7 (1 = not at all true, 4 = somewhat true, and 7 = very true). 1–3 = not true, 4–5 = somewhat true, and 6–7 = very true*.

## Discussion

This pilot study explored the effectiveness of nutrition education tools in young adolescents comparing an educational escape game with traditional self-study learning method. The results found that both educational escape game and self-study intervention conditions showed significant improvements in short-term knowledge when comparing baseline and post-intervention nutrition knowledge assessments, though no appreciable differences were found between the two conditions.

The results of this study corroborate the findings from previous studies gamifying health education. A meta-analysis found the positive effect size of health promotional board games on knowledge large (Cohen's *d* = 0.82, 95%CI: [0.15, 1.48]) and on behavior moderate [Cohen's *d* = 0.38, 95% CI (0.07, 0.69)] (15). Much like the present study, a recent study on school-aged children that piloted the digital game “Fit Food Fun” with traditional teaching techniques found both gaming and traditional methods to be effective tools for short-term improvement in nutrition-related knowledge (20).

Unlike the present study, the ETIOBE Mates digital game was reported to have had higher improvement in nutrition-related knowledge scores among young adolescents when compared to a leaflet control condition after 2-weeks of intervention (19). A cluster-randomized controlled trial of 20 schools studied the effects of the health promotional board game, Kaledo, in school-aged children when exposed to short gameplay once a week for 20-weeks. Regarding nutrition knowledge assessment, researchers found sustained improvement 6-months and 18-months post intervention compared to a non-treatment control among adolescents in middle school (21). These controlled intervention trials suggest that gamed-based learning may be able to provide additional learning opportunities when reinforcing traditional teaching methods.

The results from this pilot study begin to address gaps in the literature by investigating escape games as educational tools in school-aged children and as nutrition intervention tools. There are only a few studies measuring the effectiveness of educational escape games, none of which study adolescents or children. Preliminary results presented in a conference observed the Zombie Attack educational escape game to increase measured nutrition-related knowledge in 228 adults across five countries (26). A diabetes management escape game for pharmacy students (*n* = 74) found significant improvement in knowledge comparing pre and post assessment scores (25). A more recent escape game trial with nursing students found improvement in most knowledge questions after gameplay, though statistical significance was not calculated (27). On the other hand, a study on 63 pharmacy students reported that participants had a lower score in knowledge after the post intervention assessment, even though 96% stated they felt the game facilitated learning and improved their clinical skills. However, the authors hypothesized that the observed decrease in knowledge was due to misplaced incentive, as pre-assessment results weighed for about a third of the class grade and post-assessment results were unsubstantial (28).

The participants of the educational escape game condition reported to having enjoyed the game and generally perceived the game as beneficial and of value for them. This coincides with all descriptive studies on escape games, which report tremendously positive feedback for enjoyment and their perceived potential to facilitate learning and motivation (27, 29, 33–38). It is theorized that the increased intrinsic motivation with gaming stems from immersion, high concentration, challenges and accomplishments throughout the gameplay experience (17, 22), which are quintessential qualities of escape games. Teaching tools that stimulate intrinsic motivation and are enjoyable have the potential to facilitate further learning, which is particularly useful in school settings where various educational approaches can be utilized. Moreover, there is evidence that active modes of education (e.g., interactive game-based learning) are more effective than passive modes (e.g., self-study) and that active education is generally more suitable for teaching (39).

The intervention content had a focus on promoting healthy and sustainable plant-based protein sources, which may have influenced the overall positive gain in knowledge. A study on adolescents across Europe observed interventions that promoted healthy habits were more accepted than those that discouraged unhealthy dietary habits (40). The present intervention attentively promoted and educated participants on healthful plant-based protein sources and their role in environmental sustainability, which can be more impactful to youth motivation in comparison to future compromised health status. A double-blind randomized controlled trial found that, in eighth graders, autonomy, and social justice fueled healthful choices more than personal health implications (41). This indicates that interventions focusing on sustainability and planetary health may be most appropriate for peaking motivation and eliciting behavior changes in adolescents. Furthermore, it suggests that the dietary intervention programs for adolescents should differ compared to adults, with less emphasis on personal health gain and more on providing opportunity for autonomous choices and increased environmental and societal health.

Subdomain analysis of nutrition knowledge topics found that the educational escape game condition did not significantly improve knowledge in protein-related questions, where the self-study intervention condition did. Considering the intervention materials had a focus on plant-based protein sources, the effectiveness of internalizing key messages may be muddled in gameplay in comparison to self-study methods, where the main messages are readily identifiable. Prospective educational escape game designers should consider repetition of highlighted objectives from the overarching themes throughout the gameplay. Future studies on educational escape games should investigate if the simulated panic in storyline hinders the internalization of key messages. Moreover, learning mechanisms should be studied in more detail to understand which types of puzzles result in the most effective learning outcomes.

Strengths of this study include the binational and comparative design, the random allocation by class, and the homogeneity between intervention conditions. Questionnaires were adapted from previously validated questionnaires and, though not validated for the current study population, the nutrition knowledge questionnaire produced similar baseline results to a questionnaire validated for 12–14 year old adolescents aged 12–14 in Italy (14). Bias regarding classroom heterogeneity was alleviated by collecting data for both interventions from every classroom. Therefore, any baseline differences between classrooms were likely equally distributed between the intervention conditions. In addition, the escape game Zombie Attack had been pretested on adults and the results were used to adjust the prototype and remove inconsistencies. Likewise, the escape game has already shown positive results for effectiveness in knowledge assessment with adult populations in preliminary pilot studies (26).

When compared to other game-based interventions, a general limitation to an educational escape game is that the intervention can only be administered once, due to the unrepeatable nature of escape games. This pilot intervention study has many additional limitations. For instance, these intervention results cannot be extrapolated to long-term gains in nutritional knowledge, since only results from short-term knowledge change were measured. This pilot sample size was rather small in number, with 18% of participants with missing the baseline or intervention data. Initially, teachers were invited to participate in the study, introducing a selection bias, as participating classrooms may have more motivated teachers inclined to trial innovative learning techniques. Likewise, selection bias may also be present in students and parents, as only those with written parental consent were permitted to participate. Though participants were advised not to discuss interventions during the intervention day, it is not possible to completely rule out cross-contamination outside the monitored intervention conditions. Moreover, since UK class participants conducted the interventions in the same area and time, cross-contamination between and within intervention conditions was possible.

Country specific differences are also noteworthy. The Finnish study sample was about a year older than the intervention mean and the participants all attended school with a physical-education focus, which likely contributed to the higher mean assessment results in Finland compared to UK. Unlike in Finland, the UK sample consisted mostly of girls (98%) due to the proportion of girls in the participating classrooms, and, thus, strongly skewed the overall study population to be overrepresented by girls (81%). Future trials should consider a more gender-equivalent study population, as young adult and adolescent females may have more interest and motive for healthy eating compared to males (42). Likewise, the participants from Finland may not have been representative of typical baseline nutrition knowledge for that age, as they attended a school specialized in physical education and, therefore, may have more knowledge on health-related topics. Lastly, it is notable that nutrition knowledge may vary due to cultural differences between Finland and the UK. However, the lack of difference in overall gained knowledge between the two countries suggests that these underlying differences in population should not affect potential for positive knowledge improvement.

This binational pilot study found the use of an educational escape game as sufficient as traditional self-study methods for teaching school-aged children about nutrition. Both intervention conditions showed improvement in nutritional knowledge between baseline and post-interventions. These findings provide evidence that game-based teaching techniques can offer comparable intervention strategies for improving diet-related knowledge compared to traditional, self-study teaching methods. This study also found educational escape games to be an effective and encaptivating tool for nutrition education in school-aged children. Since this pilot study was unable to assess behavioral changes and long-term retention of gained knowledge, future intervention trials are needed to evaluate the potential benefits of educational escape games in both nutrition knowledge and dietary behavior change.

## Data Availability Statement

The raw data supporting the conclusions of this article will be made available by the authors, without undue reservation.

## Ethics Statement

The studies involving human participants were reviewed and approved by the Ethical Review Board in the Humanities and Social and Behavioral Sciences of the University of Helsinki (statement 10/2019) and the University Research Ethics Committee of the University of Reading (statement 19/20). Written informed consent to participate in this study was provided by the participants' legal guardian/next of kin.

## Author Contributions

NM, HV, and MF acquired the data and conducted the research. AA analyzed the data and drafted the manuscript. NM, HV, MM, MG, and MF critically revised the manuscript for important intellectual content. All authors read and approved the final manuscript.

## Conflict of Interest

The authors declare that the research was conducted in the absence of any commercial or financial relationships that could be construed as a potential conflict of interest.
